# Rapid and visual detection of porcine deltacoronavirus by recombinase polymerase amplification combined with a lateral flow dipstick

**DOI:** 10.1186/s12917-020-02341-3

**Published:** 2020-05-07

**Authors:** Xiang Gao, Xinsheng Liu, Yongguang Zhang, Yanming Wei, Yonglu Wang

**Affiliations:** 1 College of Veterinary Medicine, Gansu Agricultural University, Lanzhou, 730070 China; 2 State Key Laboratory of Veterinary Etiological Biology, Key Laboratory of Animal Virology of the Ministry of Agriculture, Lanzhou Veterinary Research Institute, Chinese Academy of Agricultural Sciences, Lanzhou, 730046 China

**Keywords:** *Porcine Deltacoronavirus*, Recombinase polymerase amplification, Lateral flow dipstick, Rapid diagnosis, Visual detection

## Abstract

**Background:**

*Porcine Deltacoronavirus* (PDCoV) is a newly emerging *Coronavirus* that was first identified in 2012 in Hong Kong, China. Since then, PDCoV has subsequently been reported worldwide, causing a high number of neonatal piglet deaths and significant economic losses to the swine industry. Therefore, it is necessary to establish a highly sensitive and specific method for the rapid diagnosis of PDCoV.

**Results:**

In the present study, a highly sensitive and specific diagnostic method using recombinase polymerase amplification combined with a lateral flow dipstick (LFD-RPA) was developed for rapid and visual detection of PDCoV. The system can be performed under a broad range of temperature conditions from 10 to 37 °C, and the detection of PDCoV can be completed in 10 min at 37 °C. The sensitivity of this assay was 10 times higher than that of conventional PCR with a lower detection limit of 1 × 10^2^ copies/µl of PDCoV. Meanwhile, the LFD-RPA assay specifically amplified PDCoV, while there was no cross-amplification with other swine-associated viruses, including *Porcine epidemic diarrhea virus* (PEDV), *Transmissible gastroenteritis virus* (TGEV), *Porcine kobuvirus* (PKoV), *Foot and mouth disease virus* (FMDV), *Porcine reproductive and respiratory syndrome virus* (PRRSV), *Porcine circovirus type 2* (PCV2), *Classical swine fever virus* (CSFV) and *Seneca valley virus* (SVV). The repeatability of the test results indicated that this assay had good repeatability. In addition, 68 clinical samples (48 fecal swab specimens and 20 intestinal specimens) were further tested by LFD-RPA and RT-PCR assay. The positive rate of LFD-RPA clinical samples was 26.47% higher than that of conventional PCR (23.53%).

**Conclusions:**

The LFD-RPA assay successfully detected PDCoV in less than 20 min in this study, providing a potentially valuable tool to improve molecular detection for PDCoV and to monitor the outbreak of PDCoV, especially in low-resource areas and laboratories.

## Background

*Porcine Deltacoronavirus* (PDCoV) is an enveloped, single-stranded, positive-sense RNA virus and a member of the genus *Deltacoronavirus* in the family *Coronaviridae* that causes diarrhea, vomiting, dehydration and mortality in neonatal piglets [1–3]. The full genome of PDCoV is approximately 25.4 kb in length [2] and encodes genome arrangements in the following order: 5′ untranslated region (UTR), open reading frame 1a/b (ORF1a/b), spike (S), envelope (E), membrane (M), nonstructural protein 6 (NS6), nucleocapsid (N), nonstructural protein 7 (NS7), and 3′ UTR [4]. PDCoV was first reported in pig feces collected in Hong Kong during the molecular surveillance for Coronavirus (CoVs) in avian and mammalian species in 2011 [2]. However, the first outbreak of PDCoV was announced in Ohio in 2014 [5]. Soon thereafter, PDCoV spread to several swine-producing states of the United States, causing mortality of 30 ~ 40% in neonatal pigs [6]. To date, PDCoV has been detected/ isolated in pigs across the world, including Canada [7], South Korea [8], China [9], Thailand [10], and Vietnam [11]. Additionally, PDCoV infection has become prevalent in pig farms around the world, which has caused enormous economic losses in multiple countries and remains a serious challenge to the swine industry [12, 13]. At present, PDCoV is often seen in coinfection with other viral diarrheal pathogens with very similar clinical symptoms, which makes it difficult to effectively diagnose [14]. Furthermore, there are no effective vaccines or treatments for PDCoV, and the mechanism of its pathogenesis is still largely unknown [15]. Therefore, it is essential to develop a rapid, simple and highly sensitive diagnostic method to improve the efficacy of current control programs.

Recombinase polymerase amplification (RPA) is a novel isothermal DNA amplification technology that is remarkable due to its simplicity, high sensitivity, and compatibility with multiplexing, and is used for the detection of various infectious agents [16]. The RPA amplification product can be easily detected by lateral flow dipstick (LFD), where results are rapidly obtained in a visual read-out format [16]. Using this method, the products could be exponentially amplified in 30 min at a low and constant temperature, without the need for an initial denaturation step or the use of multiple primers [17, 18]. In recent years, LFD-RPA has been used for the diagnosis of many pathogens, including *Borrelia burgdorferi* [19], *Neospora caninum* [20], *Mycobacterium tuberculosis* [21], *Francisella tularensis* [22] and *Foot-and-mouth disease virus* [23]. In the present study, we developed a LFD-RPA diagnostic method that is simple, highly sensitive and specific for the rapid and visual detection of PDCoV in clinical fecal samples from pigs. This assay greatly reduces the time for detection and dependence on the experimental instrument and generates a potential improvement in the diagnosis of PDCoV.

## Results

### Optimization of LFD-RPA conditions

**Fig. 1:**
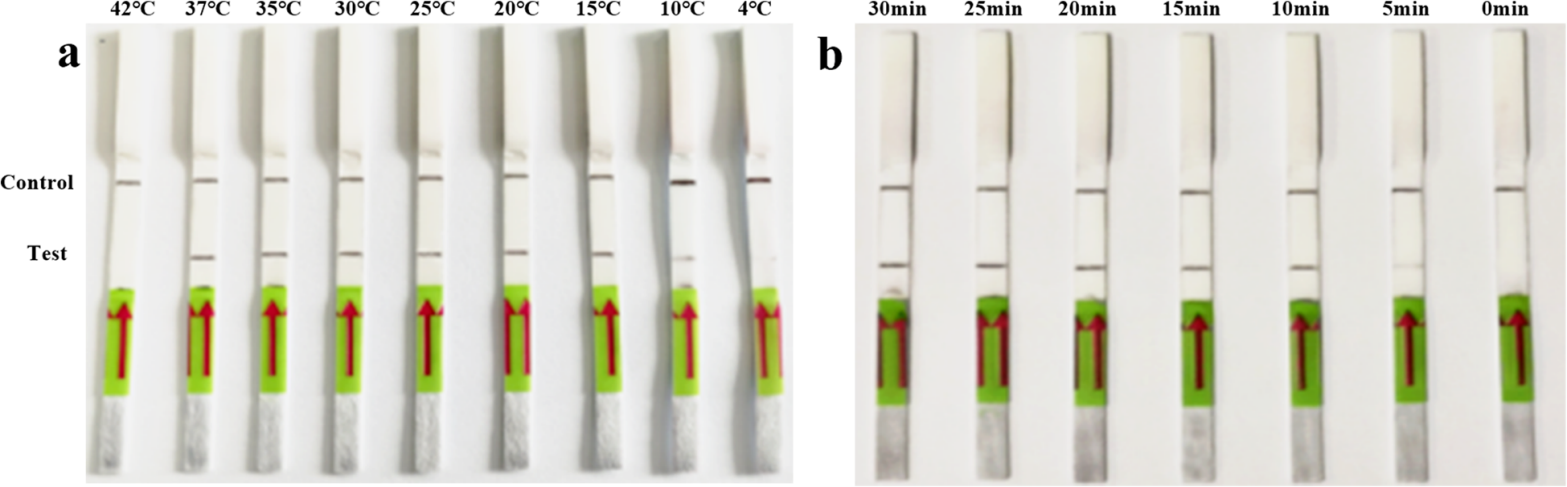
Optimization of reaction temperature and time of the recombinase polymerase amplification combined with a lateral flow dipstick (LFD-RPA) assay using 1 × 10^7^ copies/μL of recombinant plasmid DNA as a template. The top line on the lateral flow dipstick is the control line, and the next line is the test line. **a** Evaluation of different reaction temperatures from 4 to 42 °C for 20 min. **b** Evaluation of different reaction times between 0 and 30 min at 37 °C

In the detection of PDCoV by the LFD-RPA assay, an intense test band could be observed on the lateral flow dipstick with the specific primers and probe. As shown in Fig. 1a, the LFD-RPA assay works well in the wide temperature range of 10 to 37 °C. Meanwhile, a light target band was detected in as little as 5 min after incubation in the 37 °C metal bath, while there was no significant difference at 10, 15, 20, 25 and 30 min (Fig. 1b). Therefore, considering the detection specificity and efficiency, the amplification temperature and time of 37 °C and 10 min may be the best reaction conditions for detecting PDCoV by LFD-RPA.

### LFD-RPA sensitivity

**Fig. 2:**
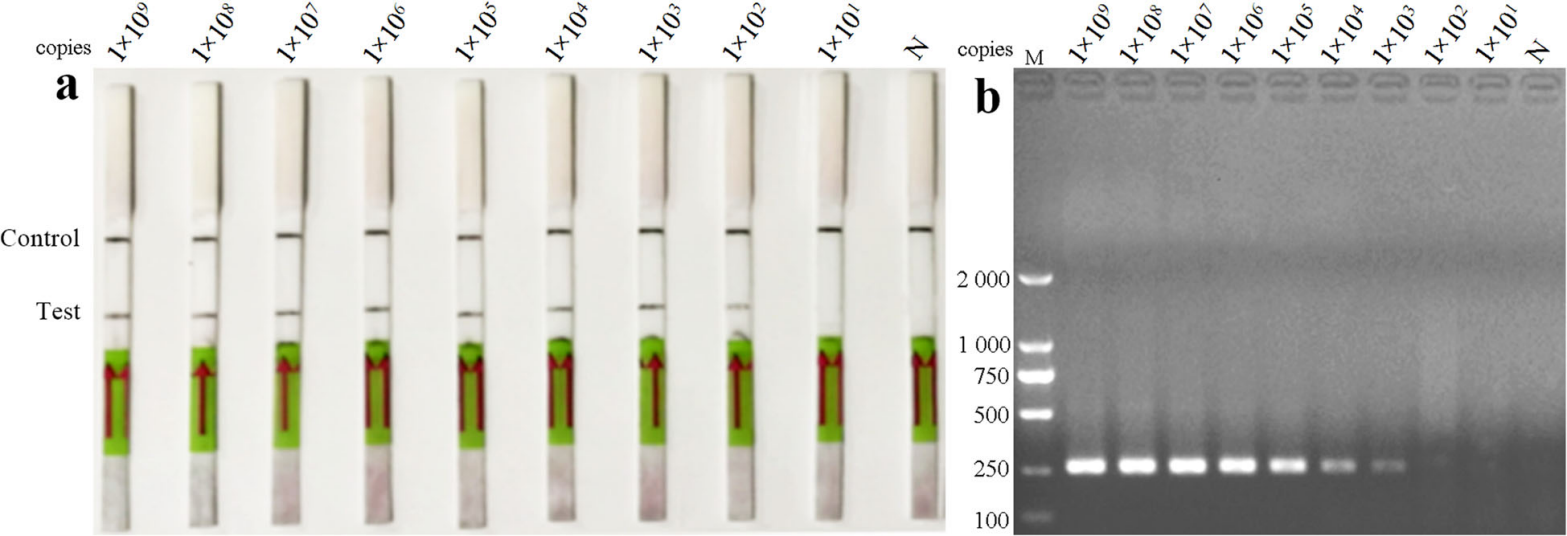
Sensitivity of the LFD-RPA assay and conventional RT-PCR using 10-fold serial dilutions of standard control plasmids at concentrations ranging from 1 × 10^9^–1 × 10^1^ copies/μL; previously tested PDCoV negative samples was used as a negative control. The top line on the lateral flow dipstick is the control line, and the next line is the test line. **a** Standard control plasmids at concentrations ranging from 1 × 10^9^–1 × 10^1^ copies/μL. **b** Standard control plasmids at concentrations ranging from 1 × 10^9^–1 × 10^1^ copies/μL (lanes 1–9), and N is a negative control. M is a DNA marker

To verify the sensitivity of LFD-RPA, a dilution series of standard plasmids ranging from 10^9^ to 10^1^ copies/μL was used. The results demonstrated that the limit of detection with the LFD-RPA was 10^2^ copies/μL, which was higher than that of conventional PCR (10^3^ copies/μL) (Fig. 2a and b).

### Detection specificity of LFD-RPA

**Fig. 3:**
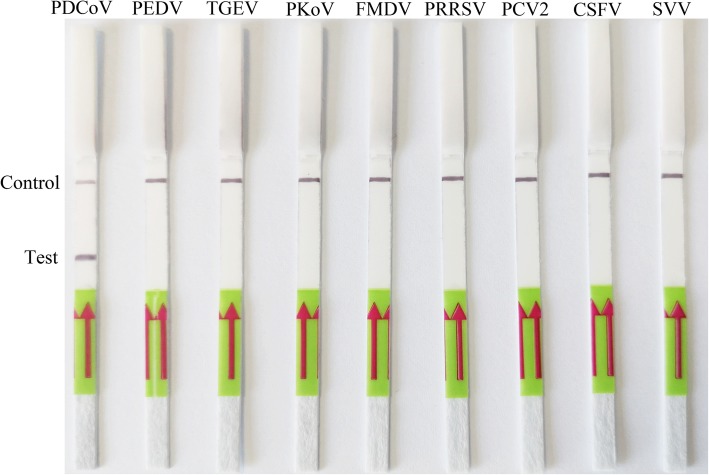
Detection specificity of the LFD-RPA assay. The top line on the lateral flow dipstick is the control line, and the next line is the test line. Specificity of the LFD-RPA assay for different species of pig viruses, including *Porcine Deltacoronavirus* (PDCoV), *Porcine epidemic diarrhea virus* (PEDV), *Transmissible gastroenteritis virus* (TGEV), *Porcine kobuvirus* (PKoV), *Foot and mouth disease virus* (FMDV), *Porcine reproductive and respiratory syndrome virus* (PRRSV), *Porcine circovirus type 2* (PCV2), *Classical swine fever virus* (CSFV) and *Seneca valley virus* (SVV)

The specificity of LFD-RPA was evaluated by swine-associated virus (PDCoV, PEDV, TGEV, PKoV, FMDV, PRRSV, PCV2, CSFV and SVV). The results showed that the primers and probe used were specific for the amplification of PDCoV (Fig. 3), and there was no cross reaction with the other tested swine-associated viruses.

### Repeatability test

**Fig. 4:**
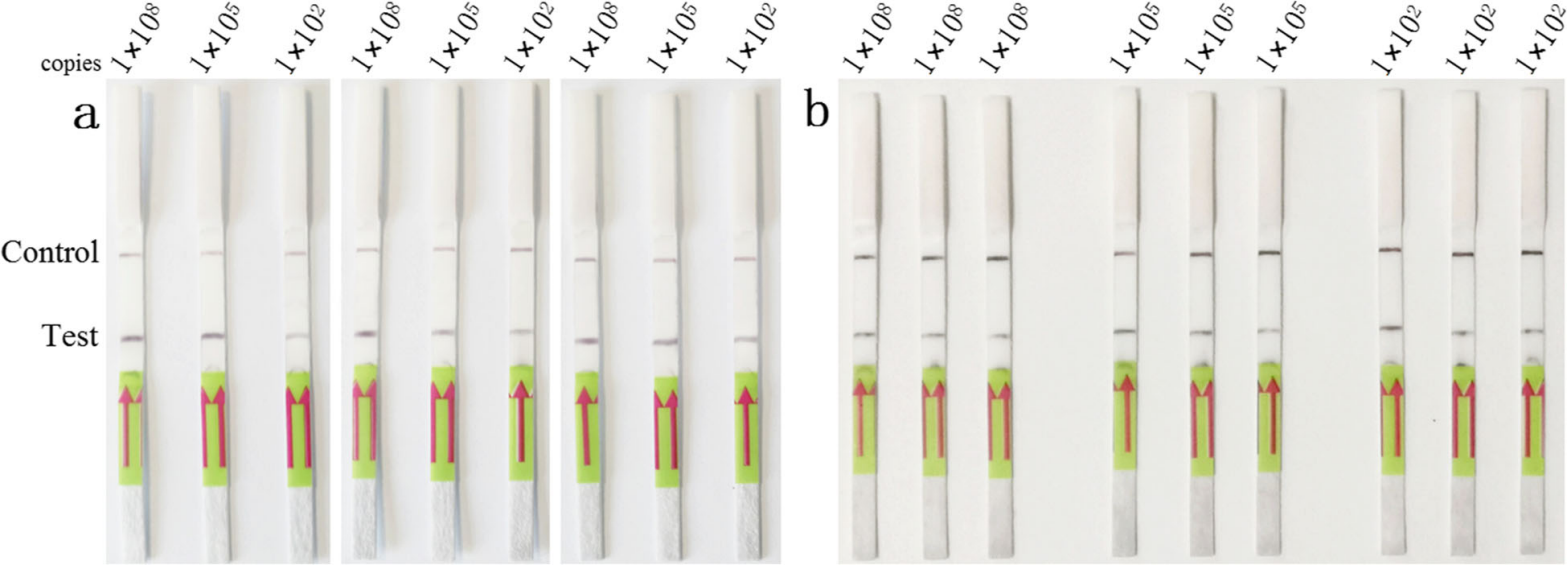
Repeatability of the LFD-RPA assay. **a** Inter-assay repeatability test: The standard control plasmids, at concentrations 1 × 10^8^, 1 × 10^5^ and 1 × 10^2^ copies/μL (lane 1–3), were detected in three rounds under the same conditions. **b** Intra-assay repeatability test: The standard control plasmids, at concentrations 1 × 10^8^, 1 × 10^5^ and 1 × 10^2^ copies/μL, repeat three tests at the same time under the same conditions. The top line on the lateral flow dipstick is the control line, and the next line is the test line

The repeatability of the LFD-RPA assay was investigated using different standard control plasmids at concentrations 1 × 10^8^, 1 × 10^5^ and 1 × 10^2^ copies/μL under the same conditions, and the assay exhibited the same results in inter- and intra-assay replicate experiments (Fig. 4).

### LFD-RPA performance compared with conventional PCR using clinical samples

In a total of 68 clinical samples from subjects exhibiting diarrhea, the detection results indicated that the positive rate of the LFD-RPA method was 26.47%, which was higher than that of the conventional PCR assay (23.53%, Table 1).

**Table 1:** Comparison of LFD-RPA and conventional PCR in clinical samples

Samples type	Number of samples	LF-RPA		Conventional PCR	
		Positive	Negative	Positive	Negative
fecal swab	48	13	35	12	36
intestinal	20	5	15	4	16
Total	68	18	50	16	52

## Discussion

The LFD-RPA assay is a rapid, highly sensitive and selective detection method, and the results are obtained in a visual read-out format, which has been explored for the detection of diverse pathogens [16]. In the current study, we developed a rapid and visual method of LFD-RPA for detecting PDCoV in clinical samples. It is much faster and more convenient than conventional RT-PCR. The LFD-RPA reaction can be accomplished in less than 20 min at 10–37 °C without any expensive high precision instrumentation. This means that the reaction can operate efficiently at body temperature without the need for special equipment [24]. In addition, the detection limit of the LFD-RPA assay was 10^2^ copies/μL, which was more sensitive than the conventional RT-PCR (10^3^ copies/μL). Likewise, Ma et al. [25] recently reported a detection limit as low as 10^2^ copies/μL of PDCoV by a real-time reverse transcription recombinase polymerase amplification (RT-RPA) assay, whose sensitivity was the same as that of LFD-RPA developed in the present study. The specific validation test indicated that the method is useful for PDCoV detection, and there was no cross-reaction with the several reference swine viruses used in the study. Moreover, the repeatability assay demonstrated that LFD-RPA has good repeatability for detecting PDCoV. Subsequently, LFD-RPA was used to detect the clinical samples to evaluate the performance. The results showed that the positive detection rate of LFD-RPA was higher than that of conventional RT-PCR, which indicating that LFD-RPA is capable of effectively and accurately detecting PDCoV. All the integrated data showed that LFD-RPA is a simple, visible, specific and sensitive tool for the rapid and accurate detection of PDCoV that is especially suitable for use in underequipped laboratories and at point-of-need facilities, which is of great significance for PDCoV control in low resource settings.

Many relevant detection methods for PDCoV infections have been carried out, such as loop-mediated isothermal amplification (LAMP), indirect enzyme linked immunosorbent assay (ELISA), RT-PCR, and real-time RT-PCR [26–29]. However, those methods inherently require the use of specialized equipment and long reaction times, thus restricting their use in rapid detection and by low-resource laboratories. Although the LAMP assay is also an isothermal amplification technique that does not require expensive equipment, optimum reaction conditions that require a specific amplification temperature were performed at 63 °C for 70 min and required five primers [26]. Furthermore, the results were visualized by adding SYBR Green I dye to the reaction tube [26]. Real-time RT-PCR and RT-PCR, which are simple methods for detecting PDCoV, have been developed with high sensitivity and specificity, but these assays are difficult to perform in resource-poor areas and are not appropriate for field detection of PDCoV [25, 30]. Recently, a rapid and accurate real-time RT-RPA was established for the detection of PDCoV [25], while observations of results require specific equipment. However, compared with previous studies, LFD-RPA was carried out at a lower temperature (10–37 °C), under a shorter time (< 20 min) and required only a pair of primers in this study. In addition, the amplification product was visible on the LF dipstick inspected by the naked eye within 10 min and by untrained personnel. Furthermore, the LFD-RPA could also be easily performed in a water bath or even using body heat in field conditions, which favors the point-of-care detection of PDCoV and those scenarios involving a lack laboratory equipment. Thus, LFD-RPA offers significant benefits, and this method could be an ideal tool to improve the diagnostic efficiency and may contribute to effective surveillance of PDCoV in epidemical areas.

## Conclusions

In summary, a highly sensitive and specific method was established to provide a more practical technique for the rapid and visual detection of PDCoV by RPA assay combined with LFD. This assay has the advantages of being simple, time-effective and inexpensive, which are comparatively applicable for underequipped laboratories as potential tools for preventing and controlling the spread of PDCoV.

## Methods

### Sample collection and viruses

A total of 68 clinical samples (48 fecal swab specimens and 20 intestinal specimens) were collected from piglets or pigs on different farms with acute diarrhea in six Chinese provinces during 2016–2017 and stored at − 80 °C. *Porcine Deltacoronavirus* (PDCoV), *Porcine epidemic diarrhea virus* (PEDV), *Transmissible gastroenteritis virus* (TGEV), *Porcine kobuvirus* (PKoV), *Foot and mouth disease virus* (FMDV), *Porcine reproductive and respiratory syndrome virus* (PRRSV), *Porcine circovirus type 2* (PCV2), *Classical swine fever virus* (CSFV) and *Seneca valley virus* (SVV) were isolated from field samples and stored in our laboratory. PDCoV, PEDV and FMDV were tested by real-time RT-PCR in the study as positive controls, while the remaining viruses were detected by conventional PCR.

### Isolation of viral RNA and synthesis of cDNA

The clinical samples were diluted 10-fold with serum-free Modified Eagle Medium (MEM, Invitrogen, USA) containing 1% penicillin-streptomycin (10,000 units/mL of penicillin and 10,000 μg/mL of streptomycin, Gibco™, USA), vortexed, and centrifuged at 3500 rpm at 4 °C for 30 min. The viral RNAs were extracted from clinical samples using the Viral RNA Mini Kit (Qiagen, Germany), and reverse transcription was performed using the avian myeloblastosis virus reverse transcriptase kits (Promega, USA) in a total volume of 25 μL according to the manufacturer’s instructions and the reverse transcription procedure was 42 °C for 1 h. All cDNA samples were stored at − 20 °C until use.

### Construction of molecular standard plasmid DNA

The nucleocapsid (N) gene of PDCoV (GenBank No: KJ481931) was selected as the targeted region of the RPA because the N gene is highly conserved among different isolates of PDCoV. The PCR amplification system was as follows: 2 μL PrimeSTAR GXL DNA polymerase (TaKaRa, Dalian, China), 10 μL 5 × PrimeSTAR GXL Buffer, 4 μL dNTP Mixture (10 μM), 2 μL of each primer N-F/N-R (Table 2), and 3 μL DNA template and 27 μL double distilled H_2_O (ddH_2_O). The temperature profile consisted of activation at 98 °C for 3 min followed by 30 cycles of PCR at 98 °C for 10 s, 60 °C for 15 s, 68 °C for 20 s and a final extension step at 68 °C for 3 min. The PCR products were purified with the PureLink™ Quick Gel Extraction and PCR Purification Combo Kit (Invitrogen, Darmstadt, Germany) and then ligated into the pUC20-T vector (TaKaRa, Dalian, China) and translated into *E. coli* DH5α cells (TaKaRa, Dalian, China). DNA concentrations were quantified using Thermo Scientific NanoDrop 2000 spectrophotometers (Thermo, Wilmington, USA). The quantity of DNA copies was calculated by the formula: DNA copy number (copies/μL) = (M × 6.02 × 10^23^ × 10^− 9^)/(n × 660), where M is molecular weight and n is the plasmid concentration (g/μL) measured at 260 nm. Dilution of the copy number of the recombinant plasmid to 10^9^ ~ 10^1^ was performed to analyze sensitivity.

**Table 2:** The primers and probe for conventional PCR and LFD-RPA assay used in this assay

Assay	Primers and probe	Sequences 5′-3’	Amplicon size (bp)
RPA	N-F	CGTCGTAAGACCCAGCATCAAGCTCCCAAGCGGAC	257
	N-R	GATTATGCTGTACCCTCGATCGT	
LFD-RPA	LFD-F	CGTCGTAAGACCCAGCATCAAGCTCCCAAGCGGAC	257
	LFD-R	Biotin-GACTGTGATTGAGTAGGAGAAGGTAAGGGTAATTG	
	LFD-Probe	FITC-GTCGGCTCTGGAGACACTGAGAAGACGGGT (THF)ATGGCTGATCCTCGCATCA-C3-Spacer	

### Primer and probe design

The N-specific primers and a probe for LFD-RPA were designed using Primer 3 V.4.0.0 (http://bioinfo.ut.ee) according to the TwistDx guidelines, with the reverse primer labeled with biotin at the 5′ end. The primers and probe were synthesized by Takara company (Dalian, China). Detailed information on PDCoV LFD-RPA is listed in Table 2.

### Development of LFD-RPA assays

The RPA assay was performed as described in the operation manual of the TwistAmp nfo Kit (TwistDx, Cambridge, UK). Briefly, each amplification mixture of the RPA assay with the following total volume of 50 μL was as follows: 29.5 μL 1 × rehydration buffer, 2.4 μL each primer LFD-F (10 μM)/LFD-R (10 μM), 0.6 μL LFD-probe (10 μM), 2 μL DNA template and 10.6 μL ddH_2_O. For each sample, a total of 47.5 μL of the mixture was added to dissolve the freeze-dried enzyme pellet, and the reaction started with 2.5 μL of 280 mM magnesium acetate. Then, the tubes were placed in a 37 °C metal bath with constant shaking at 300 rpm for 20 min [31]. Milenia Genline HybriDetect LFD strips (Milenia Biotec, Giessen, Germany) are a simple tool for quantitative, semi-quantitative and to some extent quantitative monitoring [32], used in this study to directly observe the results of RPA products. After incubation, the RPA products were diluted 1:10 with the assay buffer (supplied by the kit) and then LFD strips were inserted vertically into 200 μL of diluted product at room temperature with the final result read within 10 min. A positive result clearly showed a test line and control line, while the negative reactions only generated a control line.

### Optimization of LFD-RPA conditions

The reaction temperature and time are two key parameters of LFD-RPA, which are optimized to improve detection efficiency. The LFD-RPA assay was tested on 1 × 10^7^ copies/μL of standard plasmid DNA as a template, and the optimum reaction was determined at different reaction temperatures: 4, 10, 15, 20, 25, 30, 35, 37 and 42 °C for 20 min. After identifying the optimal temperature, the reaction was performed for 0, 5, 10, 15, 20, 25 and 30 min at 37 °C to determine the minimum amplification time required for the reaction.

### Sensitivity of LFD-RPA assay

To determine the sensitivity of the LFD-RPA assay, 10-fold serial dilutions were performed to obtain a gradient of standard plasmid DNA from 1 × 10^9^ to 1 × 10^1^ copies/μL with nuclease-free water. Conventional PCR was used to compare sensitivity with the LFD-RPA assay, and previously tested PDCoV negative samples was used as a negative control. Therefore, 1 μL diluted DNA was used as a template for LFD-RPA and then incubated at 37 °C for 10 min with 300 rpm constant shaking. The diluted DNA was also used in the conventional PCR reaction.

### Specificity of LFD-RPA assay

Other common swine-associated viruses, such as PDCoV PEDV, TGEV, PKoV, FMDV, PRRSV, PCV2, CSFV and SVV were used to assess the specificity of the LFD-RPA assay under optimal conditions.

### Repeatability test

To confirm the repeatability of the LFD-RPA assay, standard control plasmids at concentrations 1 × 10^8^, 1 × 10^5^ and 1 × 10^2^ copies/μL were used. For inter-assay repeatability test, three independent tests were performed on three different days under the same conditions. For intra-assay repeatability test, the same plasmid were tested three times at the same time under the same conditions.

### LFD-RPA performance compared with conventional PCR using clinical samples

A total of 68 clinical samples (48 fecal swab specimens and 20 intestinal specimens) were collected from piglets or pigs on different farms with acute diarrhea in six Chinese provinces during 2016–2017. All samples were then examined for the presence of PDCoV by conventional PCR and LFD-RPA to evaluate the detection efficiency of both assays. The conventional PCR volume 20 μL system as follow: 1 μL PrimeSTAR GXL DNA polymerase (TaKaRa, Dalian, China), 4 μL 5 × PrimeSTAR GXL Buffer, 2 μL dNTP Mixture (10 μM), 1 μL of each primer N-F/N-R (Table 2), and 1 μL DNA template and 10 μL double distilled H_2_O (ddH_2_O). The temperature profile consisted of activation at 98 °C for 3 min followed by 30 cycles of PCR at 98 °C for 10 s, 60 °C for 15 s, 68 °C for 40 s and a final extension step at 68 °C for 3 min. Meantime, positive and negative controls were set up in PCR reaction and the positive bands of clinical samples were recovered for sequencing. The LFD-RPA assays were performed the same as those mentioned above. For each sample, the reaction was tested three times to determine whether a sample was positive or negative.

## Data Availability

The datasets used and analyzed in the current study are available from the corresponding author upon reasonable request**.**

## Abbreviations

PDCoV: Porcine deltacoronavirus
PEDV: Porcine epidemic diarrhea virus
TGEV: Transmissible gastroenteritis virus
PKoV: Porcine kobuvirus
FMDV: Foot and mouth disease virus
PRRSV: Porcine reproductive and respiratory syndrome virus
PCV2: Porcine circovirus type 2
CSFV: Classical swine fever virus
SVV: Seneca valley virus
LFD-RPA: Recombinase polymerase amplification combined with a lateral flow dipstick
RT-RPA: Reverse transcription recombinase polymerase amplification
RT-PCR: Reverse transcription polymerase chain reaction
UTR: Untranslated region
ORF: Open reading frame
CoVs: Coronavirus
MEM: Modified Eagle Medium
LAMP: Loop-mediated isothermal amplification
ELISA: Enzyme linked immunosorbent assay
